# Frameless x-ray-based lead re-implantation after partial hardware removal of deep brain stimulation system with preservation of intracerebral trajectories

**DOI:** 10.1007/s00701-021-04807-1

**Published:** 2021-03-23

**Authors:** Vesna Malinova, Dariusz J. Jaskólski, Rafal Wójcik, Dorothee Mielke, Veit Rohde

**Affiliations:** 1 Department of Neurosurgery, Georg-August-University, Robert-Koch-Straße 40, 37075 Göttingen, Germany; 2 Department of Neurosurgery and Neurooncology, Barlicki University Hospital, Medical University of Lodz, Lodz, Poland

**Keywords:** Deep brain stimulation, Hardware dysfunction, Hardware infection, Frameless lead replacement

## Abstract

**Background:**

Deep brain stimulation (DBS) is an established treatment for patients with medical refractory movement disorders with continuously increasing use also in other neurological and psychiatric diseases. Early and late complications can lead to revision surgeries with partial or complete DBS-system removal. In this study, we aimed to report on our experience with a frameless x-ray-based lead re-implantation technique after partial hardware removal or dysfunction of DBS-system, allowing the preservation of intracerebral trajectories.

**Methods:**

We describe a surgical procedure with complete implant removal due to infection except for the intracranial part of the electrode and with non-stereotactic electrode re-implantation. A retrospective analysis of a patient series treated using this technique was performed and the surgical outcome was evaluated including radiological and clinical parameters.

**Results:**

A total of 8 DBS-patients with lead re-implantation using the frameless x-ray-based method were enrolled in the study. A revision of 14 leads was performed, whereof a successful lead re-implantation could be achieved without any problems in 10 leads (71%). In two patients (one patient with dystonia and one patient with tremor), the procedure was not successful, so we placed both leads frame-based stereotactically.

**Conclusions:**

The described x-ray-based technique allows a reliable frameless electrode re-implantation after infection and electrode dysfunction and might represent an efficient alternative to frame-based procedures for lead revision making the preservation of intracerebral trajectories possible.

## Introduction

Deep brain stimulation (DBS) is an increasingly used neuromodulation technique for treatment of medical refractory movement disorders, epilepsy, and other neurological and psychiatric diseases [4, 8, 15, 17, 24, 25]. The treatment efficacy substantially depends on the accurate lead placement within small target nuclei in the basal ganglia. Frame-based stereotactic techniques represent the gold standard for a precise lead positioning, which have been previously demonstrated to be superior to their frameless counterparts [19]. However, the absolute difference in error between the frame-based and frameless techniques was small, assuming that frameless techniques might serve as a feasible alternative to frame-based approaches leading to shorter procedure time and hence to higher patient comfort during the operation [6, 19]. Although, DBS-systems are implanted with the aim of providing permanent treatment, several early and late hardware-related complications might require revision operations with partial or even complete hardware removal [1–3, 9, 17, 23]. One of the most common complications after DBS-surgery is certainly the infection with a reported rate in the literature of up to 15% [1, 17, 18, 20]. Other possible reasons for recurrent operations are lead migration, loss of stimulation effect due to electrode dysfunction, or cable fracture [14, 16]. Currently, no standardized recommendations exist considering the management of complications after DBS-surgery and the re-implantation after partial hardware removal due to complications. In this work, we aim to report our experience with frameless x-ray-based lead re-implantation after partial hardware removal for infection or dysfunction of DBS-system along the pre-existing trajectory by taking advantage of the gliotic scarring surrounding the electrode. This might represent a surgical approach making the preservation of the intracerebral trajectories possible. Furthermore, we evaluated the common practice in managing complications after DBS-system implantation requiring partial hardware removal in large-volume stereotactic neurosurgical departments in Germany by conducting a short survey.

## Materials and methods

### Patient population

A retrospective analysis of DBS-patients undergoing revision operation with intracerebral lead re-implantation after removal of the implanted pulse generator (IPG) and the extension wires due to local infection while leaving the intracerebral leads in situ, in two institutions (Göttingen and Lodz), was performed. Additionally, DBS-patients with lead revision due to electrode dysfunction (loss of stimulation effect or cable fracture) were reviewed and included into the analysis of this study. Due to the retrospective nature of the study, informed consent of the included patients was waived.

### Description of the surgical technique

**Fig. 1:**
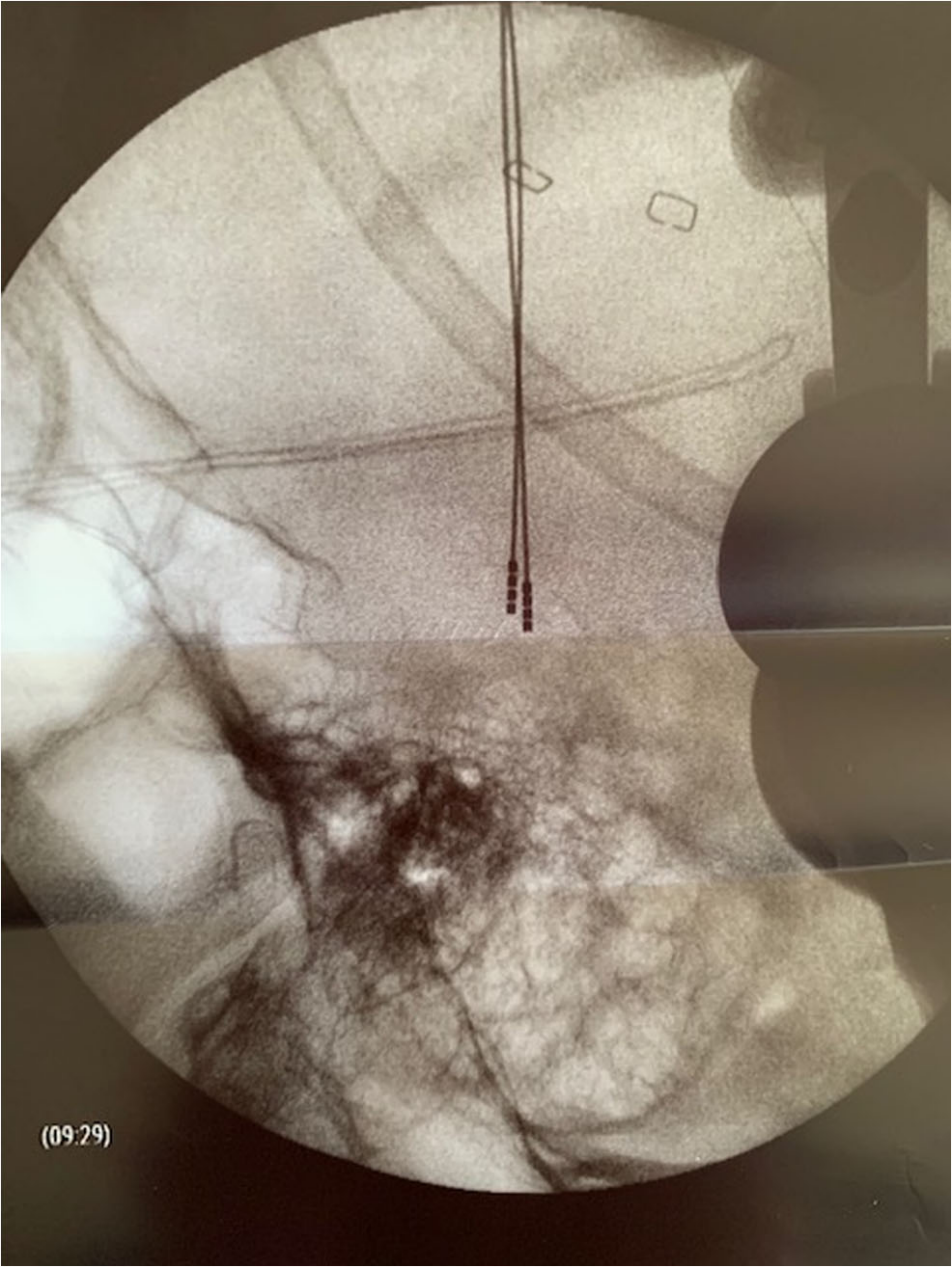
Lateral fluoroscope with depiction of the intracerebral leads

**Fig. 2:**
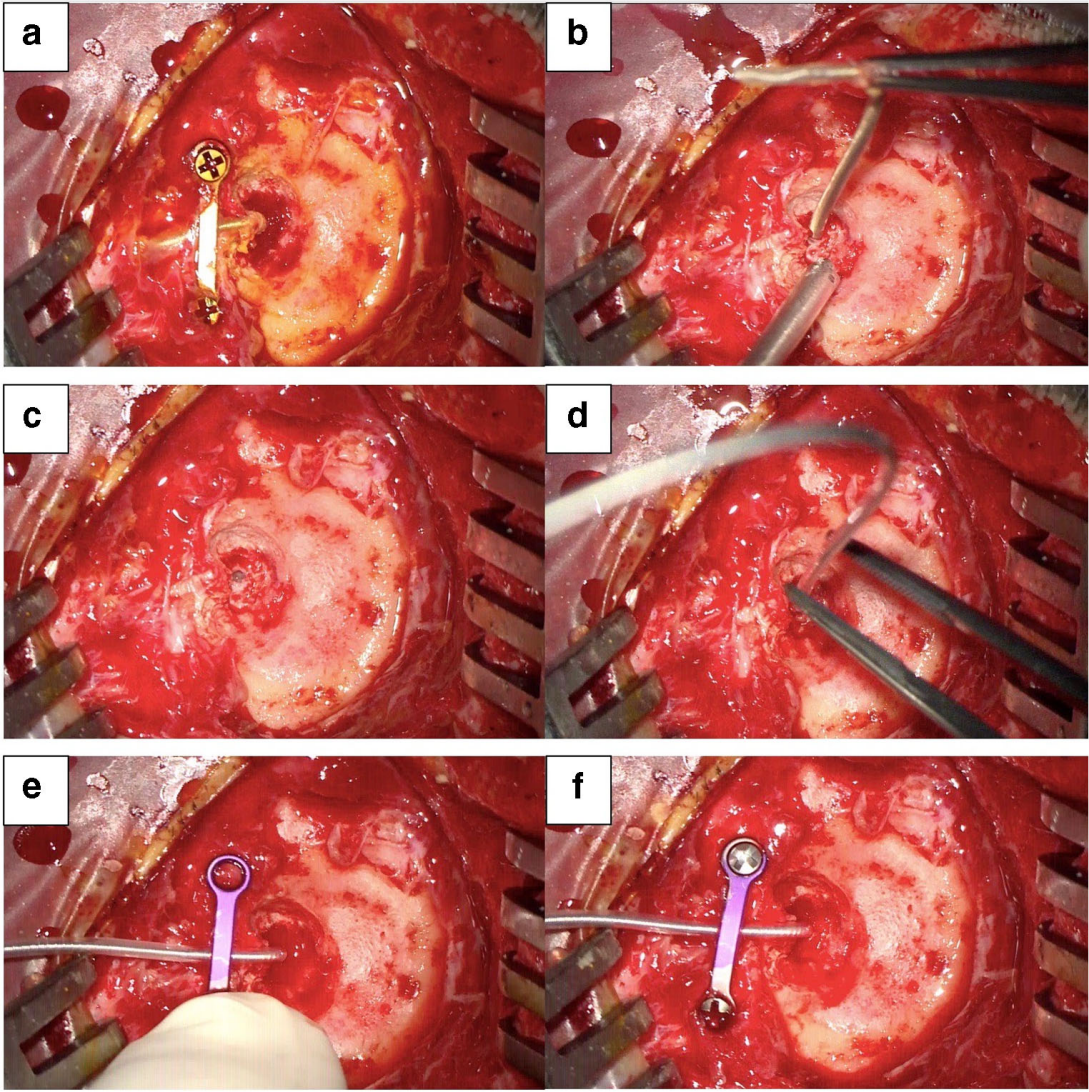
Depiction of the surgical steps. **a** Frontal skin incision is re-opened, and the lead is exposed under microscope. **b** Removal of the lead after the lead path and its tip are marked on the screen of the fluoroscope. **c** Visualization of the brain entry point. **d** The new electrode is slowly pushed forward using the existing pathway of the initial lead after removal of the guide wire of the new electrode. **e** + **f** Fixation of the new electrode with a titanium miniplate after confirming a correct position under lateral x-ray

During the DBS-system removal due to hardware infection at the connector site, all components of the DBS-system are removed except for the intracerebral leads, which are cut approximately 2 cm distal to the brain entry point and fixed with a titanium miniplate. Following a minimum of 6-week antibiotic therapy and uneventful wound healing, the procedure for electrode re-implantation is scheduled. In general anesthesia, the patient’s head is fixed in the Mayfield clamp in a supine position. A C-arm unit is positioned for lateral fluoroscopy and clear depiction of the intracerebral leads. In order to facilitate a visualization of the entire length of the lead, the patient’s head is rotated to the contralateral site allowing a strict vertical lead position. The intracerebral lead path and its tip are marked on the screen of the fluoroscope (Fig. 1). The frontal skin incision is re-opened, and a careful preparation of the lead is carried out under a microscope. Repeated lateral radiograms help to detect/rule out a change of the electrode depth as well as of the C-arm position. Then, the lead is removed, and the brain entry point is clearly identified. The guide wire of the new lead is removed. Then, the lead is slowly pushed forward using the existing pathway of the initial lead. The guide wire of the new electrode should be removed before the attempt of electrode re-positioning is made, thereby reducing the risk that the new electrode leaves the path of the initial electrode. While slight pushing of the slack electrode is necessary, no attempt should be made to overcome any resistance. Lateral x-ray is required to reassure positioning of the new electrode with any deviation into the correct depth. After reaching the target position, the new lead is fixed with either a commercially available burr-hole cap or again with miniplates. The distal electrode ending was placed in a retro-auricular pocket. If necessary, the procedure was repeated on the other side. In a final step, the electrode ending(s) are connected to new extension lead(s) and impulse generator. The consecutive surgical steps are depicted in Fig. 2. In case that the electrode re-positioning along the existing trajectory failed, the cut electrodes should be explanted, and a frame-based stereotactic procedure should be planned for lead placement.

### Surgical outcome

We evaluated the intraoperative workflow and documented whether the procedure could be performed successfully or not. The postoperative CT scan was fused with the preoperative lead trajectory, and differences between both trajectories were measured if present. Information about the clinical improvement after the lead re-implantation or re-occurrence of infection was gathered after reviewing the medical follow-up records of the patients.

### Survey in large-volume stereotactic neurosurgical departments in Germany

Furthermore, we carried out a short survey in high-volume stereotactic neurosurgical departments in Germany in order to gather information about the management of complications after DBS-surgery, especially, concerning the surgical techniques used to the re-implantation of intracerebral leads after partial removal of the DBS-system. The following questions were part of the survey: (1) Do you have an established standard operating procedure for the management of complications after DBS-surgery? (2) How do you proceed in case of infection of DBS-system? (3) Which technique do you use in case of revision of the cerebral leads? (4) Do you use the O-arm for the placement or re-positioning of cerebral leads for DBS?

### Statistical analysis

The statistical analyses were performed by means of the GraphPad Prism software (version 8, GraphPad Software, San Diego, CA, USA). Descriptive statistics was used for the assessment of the patients’ characteristics.

## Results

### Patient population

We identified 8 patients (5 patients were treated in Göttingen and 3 patients in Lodz), who complied with the inclusion criteria of the study. The mean age was 52.7 years (SD 9.9 range 42–68 years). In all patients, a Medtronic DBS-system (Medtronic GmbH, Meerbusch, Germany) was implanted. The patients’ characteristics, reasons for revision operation, and the follow-up duration are presented in Table 1.

**Table 1:** Patients’ characteristics

Patients	Sex	Age (years)	Diagnosis	DBS target	Revision indication	Infection site	Time interval from implantation to revision (months)	Follow-up since revision (months)
1	M	52	Tremor	VIM	Infection	IPG/ connector	3	49
2	M	68	Tremor	VIM	Dysfunction	/	120	20
3	M	48	Parkinson	STN	Infection	IPG/connector	20	21
4	M	62	Parkinson	STN	Infection	Connector	9	17
5	F	62	Dystonia	GPI	Infection	Connector	11	16
6	F	43	Parkinson	STN	Lead rupture	/	48	37
7	M	45	Parkinson	STN	Dysfunction	/	84	6
8	M	42	Epilepsy	ATN	Lead rupture	/	39	58

*VIM* ventral intermedial thalamic nucleus, *STN* subthalamic nucleus, *GPI* globus pallidus internus, *ATN* anterior thalamic nucleus, *IPG* implanted pulse generator

### Surgical outcome

**Fig. 3:**
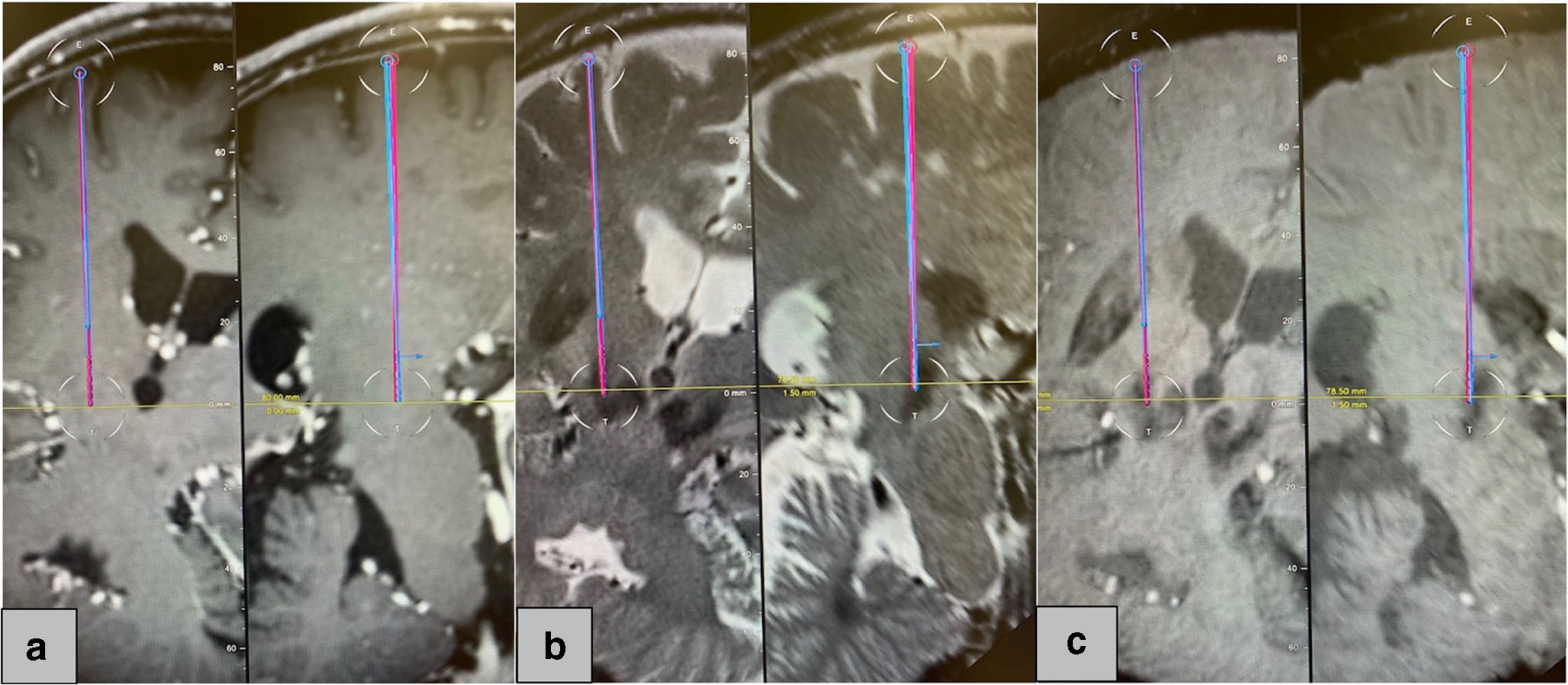
Postoperative lead localization and fusion (by means of the Brainlab® software Elements) with the preoperative MRI and the CT scan after the first lead implantation visualized on the T1 sequence after gadolinium (**a**), T2 sequence (**b**), and SWI (susceptibility weighted imaging) sequence (**c**). The initial lead position is shown as red trajectory and the lead position after the lead re-implantation as blue trajectory

A total of 14 leads were revised, whereof a successful lead re-implantation could be achieved without any problems in 10 lead revision (71%). In two patients (one patient with dystonia and one patient with tremor), it was not possible to place the lead along the same trajectory, so we decided to place both leads using a frame-based stereotaxy. No surgical complications (hemorrhage or recurrent infection) occurred in the revised patients. After the fusion of pre- and postoperative CT scan, we found no deviation of the postoperative lead position compared to the preoperative lead trajectory in all patients with successfully re-implanted electrodes. An example of both lead trajectories after the fusion using the Brainlab® software Elements (Brainlab AG, Munich, Germany) is shown in Fig. 3. An improvement in clinical symptoms could be observed in all patients after starting the stimulation. The stimulation effect was comparable with the clinical status before the partial hardware removal. The mean follow-up duration after the revision operation was 28 ± 18 months (range 6 to 58 months). None of the patients developed recurrent infection during the follow-up period.

### Survey results

We received answers of five large-volume stereotactic neurosurgical departments in Germany with the following results: (1) All departments are following the approach of preservation of the cerebral electrodes, whenever possible provided the burr hole is not affected by the infection; (2) all departments perform long-term antibiotic treatment after partial hardware removal; (3) the re-implantation is usually planned 2–3 months after partial hardware removal; (4) the cerebral lead re-implantation is performed frame-based stereotactically, whenever a revision of the cerebral electrodes is required; (5) none of the departments uses intraoperative CT or MRI for intracerebral lead placement or re-positioning for DBS.

## Discussion

In this study, we reported on our experience with frameless x-ray-based re-implantation of DBS-leads along the pre-existing trajectories, which could be successfully performed in 71% of all included cases. We proposed cutting the initial electrode at the burr-hole level, followed by a replacement of the cut electrode after eradication of the infection using lateral x-ray and taking advantage of the scarring which surrounds the electrode path, making the preservation of intracerebral trajectories possible. This procedure is indicated in patients with hardware infection at the retro-auricular connector site or in patients with skin erosion along the subcutaneous electrode path. The procedure cannot be performed in patients with highly purulent infection at the burr-hole site and if intracerebral lead infection is assumed.

### Glial scar development after initial DBS-lead implantation

A possible complication of this procedure is a lead deviation from the initial trajectory during the positioning of the new lead. The presence of a gliotic scar surrounding the electrodes should prevent this complication. A development of a glial scar around the cerebral DBS-leads has been described before, which is assumed to be the result of microglial inflammatory response to the leads [22]. A fibrous sheath of 5 to 25 μm in diameter was found in the majority of postmortem histologically analyzed cases with implanted DBS-system. Acute tissue reaction such as reactive astrogliosis was seen in 78% of cases, and chronic tissue reactions such as fibrillary gliosis were detected in 73% of the analyzed cases [7]. In a postmortem analysis of DBS-patients, the severity of gliotic response differed between the cases and did not correlate with the duration of DBS [22]. Another study with postmortem pathoanatomical evaluation of brain tissue alterations after DBS, however, described astrogliosis in all patients with long-term contact to DBS-electrode and an inflammatory and foreign-body reaction with CD3-immunoreactive T-lymphocytes in 93% of cases [13]. Taking these findings into consideration, a varying degree of gliotic scar development can be assumed after the implantation of DBS-electrodes, which can partly explain the failure to re-place the new electrodes along the pre-existing brain pathway in all patients in our study, even though the leads were in situ for at least 3 months in one case and 11 months in another case. Nevertheless, the majority of patients enrolled in our study had a much longer time interval (reaching up to 10 years) between the DBS-system implantation and the lead re-implantation leading to the assumption that longer period with the DBS-leads in situ might elevate the chances of successful lead replacement using this technique. Due to the small number of patients included in this study, our findings do not allow final conclusions concerning the right time point for performing the revision operation.

### Limitations of the technique

The potential risks of the procedure include abortion of the operation, if the brain entry point is not identifiable or if electrode deviation is visualized by fluoroscopy, less optimal lead position, intracerebral hemorrhage, and infection. Pre-requisite for electrode re-positioning using this technique is the proof that the electrode position remained unchanged during the first operative step of electrode cutting and plate fixation. The patient should be informed that the positive effect on the clinical symptoms which he/she experienced after initial surgery might be less pronounced even if intraoperative x-ray suggested a similar position of the new electrode. No data so far exist concerning the failure rate. If surgery fails, another operation for electrode re-positioning, now applying again frame-based stereotactic techniques, is necessary. An advantage of the technique that we presented in this article is that the needed equipment for performing the procedure (operative microscope and C-arm) is available in every neurosurgical operating theater. Other benefits of the procedure are the short duration and the possibility to perform the surgery under general anesthesia. Nevertheless, a lateral x-ray allows for only a limited two-dimensional depiction of the electrode position. Other methods to intraoperatively facilitate a three-dimensional depiction of the lead position are intraoperative CT or MRI [5, 6, 10, 11, 21]. Although these techniques have been shown to provide high accuracy of frameless stereotactic procedures, both modalities also require special equipment, which is a big investment for the most centers and hence not widely available [12]. This was also supported by the results of our survey, which revealed that intraoperative CT or MRI is not used in large-volume stereotactic centers in Germany.

## Conclusion

Frameless x-ray-based DBS-lead re-implantation might represent a safe and efficient alternative to frame-based procedures for lead revision after partial hardware removal allowing the preservation of intracerebral trajectories. The prerequisites for a successful procedure are clearly an identifiable electrode entry point and the presence of glial scar along the pre-existing lead trajectory. The optimal time point for performing the re-implantation after the initial surgery with much high success rate has to be defined in a future study.

## Declarations

Due to the retrospective nature of the study, an informed consent of the included patients was waived.
